# Basic Lessons From India on Vaccination [Letter to the Editor]

**DOI:** 10.5041/RMMJ.10499

**Published:** 2023-04-30

**Authors:** Thorakkal Shamim

**Affiliations:** Department of Dentistry, Government General Hospital, Manjeri, India

**Keywords:** Basic lessons, India, vaccination

**To the Editor**

The personal reflections of Peter Hotez regarding the triple threats of illness, antiscience, and antiSemitism indicate a shocking state of affairs, revealing the dark and sinister element of antivaccine activism which must be surmounted.^1^ This letter addresses basic lessons on vaccination from India in a nutshell.

The present status of adult vaccination in India is promising. Adult immunization as part of the human life course approach to health services is the key factor to achieving universal health coverage as per India’s sustainable development goal agenda 2030.^2^ In recent years, India has seen an increase in vaccination coverage from 44% to 62%—a growth of 18 percentage points.^3^ The main reason for this increase may be due to an increased reach and familiarity with vaccination that included both the urban population with India’s highest-wealth quintile and the rural population, which in comparison constitutes the lowest-wealth quintile with tribal distribution.^3^ The most important factors for vaccine hesitancy were found to be the educational status of parents, income, and lack of awareness regarding the vaccine schedule—all of which were particularly evident in the North Eastern States of India.^3^

The strategies implemented in India to address the specific determinants underlying vaccine hesitancy have included: engagement of religious or other authoritative leaders to encourage vaccination within their respective communities, social mobilization, information disseminated via the media, improving vaccination convenience and access, training of healthcare workers, and effective public health education.^4^ In India, the Social Mobilization Network (SMNet), which combines evidence-based delivery and micro-planning, was established in 2002 in Uttar Pradesh, and in 2005 in Bihar to interrupt polio transmission. Their effort utilized the mass media, print materials, house-to-house dialogues, peer-support groups, and the training and mobilization of religious and other authoritative leaders.^5^ The Community Health Worker-Led Intervention for Vaccine Information and Confidence (CIVIC) Project and the Community Accountability Board (CAB) are initiatives launched to reinforce vaccine acceptance in rural settings in India.^6^ Their strategies also targeted religious and other authoritative leaders and included: (1) knowledge-sharing and enabling interactions with healthcare providers regarding vaccination; (2) addressing vaccine effectiveness issues within social gatherings and religious meetings; and (3) short video messages on the social media platforms in local vernacular, to instill vaccine awareness and trust within the community.^6^ More recently, the Indian government, under the Ministry of Health and Family Welfare (MoHFW), has launched a national health portal that provides authenticated health information regarding immunization and vaccines and serves as a health education resource for the people of India.^7^ Moreover, under the MoHFW, the government also launched an online repository of reports, updates, promotional materials, and operational guidelines and protocols aimed at helping undergraduate and postgraduate medical professionals throughout India implement vaccination programs in their institutions.^8^

The frontline health workers (auxiliary nurses and midwives, accredited social health activists, and governmental Anganwadi childcare workers) are equipped with interpersonal communication skills via the BRIDGE (Boosting Routine Immunization Demand Generation) program, aimed at reducing vaccine hesitancy and increasing immunization coverage throughout India.^9^

Clearly, an optimistic outlook on vaccination must be maintained among different public sectors in society. Strategies for spreading such a perspective on vaccination include digitized vaccination records, mandatory vaccination certificates for school admissions, vaccination screening as part of hospital admissions, and improved vaccine education strategies to reinforce vaccine trust within the population.^3^ It is high time governments incorporate vaccine related topics in school and college curricula to ameliorate the stigma regarding vaccination.

## Abbreviations

BRIDGE: Boosting Routine Immunization Demand Generation
CAB: Community Accountability Board
CIVIC: Community Health Worker-Led Intervention for Vaccine Information and Confidence
MoHFW: Ministry of Health and Family Welfare
SMNet: Social Mobilization Network
